# Supplementary material for: Genotype by Environment Interaction in Grain Iron and Zinc Concentration and Yield of Maize Hybrids under Low Nitrogen and Optimal Conditions
Source: Plants (Basel). 2023 Mar 27;12(7):1463. doi: 10.3390/plants12071463 (PMC10096665; doi:10.3390/plants12071463)
Supplement: Supplementary file 1 [file plants-12-01463-s001.zip › plants-2277701-supplementary/Supplementary file/Supplementary Tables.pdf]

Supplementary Table S1. Mean squares of grain yield, and Fe and Zn concentration for 18 maize hybrids evaluated under optimum conditions at Cedara and Potchefstroom in South Africa during 2017 and 2018.

Source of variation	DF	Fe	Zn	GY
Environments (Env)	3	118.82**	222.15**	408.28**
Replication	1	2.14	1.19	0.12
Genotypes (Gen)	17	19.85**	19.67**	19.23**
Env*Gen	51	18.22**	9.06**	4.43**
Error	71	0.51	0.75	0.73
R^2		0.97	0.96	0.97
Mean		15.34 mg kg^{-1}	20.32 mg kg^{-1}	6.53 ton ha^{-1}
LSD		0.71	0.86	0.85

*DF = degree of freedom, GY = grain yield, R^2= Coefficient of determination, LSD = least significant difference, ** P < 0.001*

Supplementary Table S2. Mean squares of grain yield, and Fe and Zn concentration for 18 maize hybrids evaluated under low N conditions at Cedara 2017, Potchefstroom 2018, Cedara 2018 and Vaalharts 2017 in South Africa.

Source of variation	DF	Fe	Zn	GY
Environments (Env)	3	268.06**	57.21**	12.91**
Replication	1	0.08	1.45	0.58
Genotypes (Gen)	17	30.42**	32.22**	2.83**
Env*Gen	51	19.80**	10.15**	1.76**
Error	71	0.47	2.28	0.27
R^2		0.99	0.89	0.90
Mean		15.89 mg kg^{-1}	18.62 mg kg^{-1}	2.61 ton ha^{-1}
LSD		0.68	0.71	0.51

*DF = degree of freedom, Gy = grain yield, R^2 = Coefficient of determination, LSD = least significant difference, ** P < 0.001*
