# Supplementary figures and images for: Genotype by Environment Interaction in Grain Iron and Zinc Concentration and Yield of Maize Hybrids under Low Nitrogen and Optimal Conditions
Source: Plants (Basel). 2023 Mar 27;12(7):1463. doi: 10.3390/plants12071463 (PMC10096665; doi:10.3390/plants12071463)

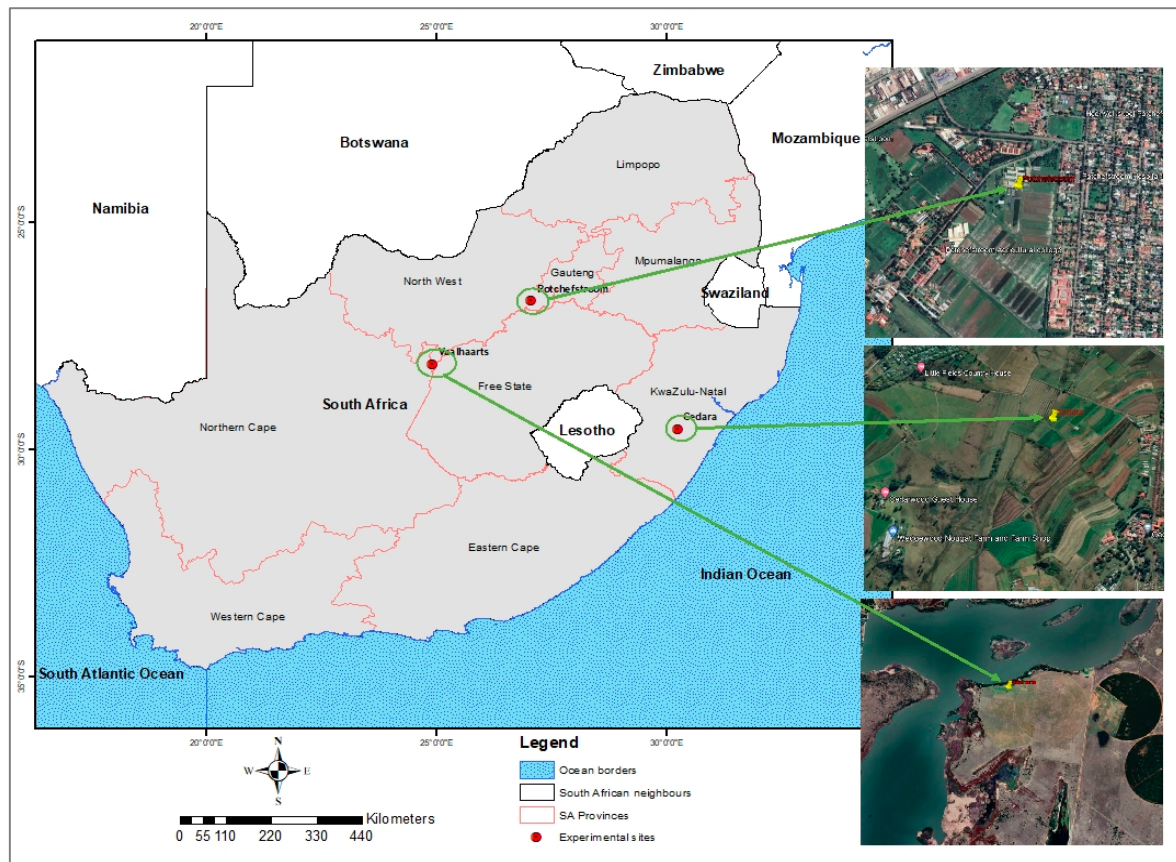

Supplementary figure 1 map of the study area

Supplement: Supplementary file 1 [file plants-12-01463-s001.zip › plants-2277701-supplementary/Supplementary file/Supplementary figure 1 map of the study area.pdf]
